# Metabolic regulation of cholestatic liver injury by D-2-hydroxyglutarate with the modulation of hepatic microenvironment and the mammalian target of rapamycin signaling

**DOI:** 10.1038/s41419-022-05450-z

**Published:** 2022-11-26

**Authors:** Xinbei Tian, Ying Wang, Ying Lu, Bo Wu, Shanshan Chen, Jun Du, Wei Cai, Yongtao Xiao

**Affiliations:** 1 Division of Pediatric Gastroenterology and Nutrition, Xin Hua Hospital, School of Medicine, Shanghai Jiao Tong University, Shanghai, 200092 China; 2 Department of Pediatric Surgery, Xin Hua Hospital, School of Medicine, Shanghai Jiao Tong University, Shanghai, 200092 China; 3 Shanghai Key Laboratory of Pediatric Gastroenterology and Nutrition, Shanghai, 200092 China; 4 Shanghai Institute for Pediatric Research, Shanghai, 200092 China

**Keywords:** Cholestasis, Metabolomics

## Abstract

Biliary atresia (BA) is a cholestatic liver disease in neonates with devastating obstructive intrahepatic and extrahepatic biliary ducts. Owing to the lack of an early diagnostic marker and limited understanding of its pathogenesis, BA often leads to death within 2 years. Therefore, this study aimed to develop early diagnostic methods and investigate the underlying pathogenesis of liver injury in BA using metabolomics. Metabolomics and organoid combined energy metabolism analysis was used to obtain new insights into BA diagnosis and pathobiology using patient samples, mice liver organoids, and a zebrafish model. Metabolomics revealed that D-2-hydroxyglutarate (D-2-HG) levels were significantly elevated in the plasma and liver of patients with BA and closely correlated with liver injuries and impaired liver regeneration. D-2-HG suppressed the growth and expansion of liver organoids derived from the intrahepatic biliary ducts. The energy metabolism analysis demonstrated that D-2-HG inhibited mitochondrial respiration and ATP synthase; however, it increased aerobic glycolysis in organoids. In addition, D-2-HG exposure caused liver degeneration in zebrafish larvae. Mechanistically, D-2-HG inhibited the activation of protein kinase B and the mammalian target of rapamycin signaling. These findings reveal that D-2-HG may represent a novel noninvasive diagnostic biomarker and a potential therapeutic target for infants with BA.

## Introduction

Biliary atresia (BA) is a severe neonatal cholestatic disease characterized by damage to the intrahepatic and extrahepatic biliary trees, obstructed bile flow, progressive fibrosis, and breakdown of the bile duct system [1, 2]. Kasai portoenterostomy (KPE) is essential for restoring bile drainage and improving the long-term survival of infants with BA. Patients with BA have the best chance of successful KPE when the operation is performed within the first 2 months [3]. Unfortunately, the early diagnosis of BA remains difficult, limiting the percentage of successful KPEs [4]. Owing to the lack of early diagnostic markers and limited understanding of liver regeneration and fibrosis, BA remains the most common cause of pediatric liver transplantation [5–7]. We recently reported that the metabolite, 2-hydroxyglutarate (2-HG), in dried blood spots (DBS) of newborns, has great potential for BA screening [8]. 2-HG consists of two enantiomeric forms, L-2-HG and D-2-HG [9], first described in 1868 by the German biochemist Karl Heinrich Ritthausen. 2-HG has not attracted much interest until recently, and its physiological function has been described [10]. Accumulating evidence suggests a strong association between 2-HG and epigenetics, signaling transduction, and other cellular processes, including the inhibition of α-ketoglutarate-dependent dioxygenases [11], reduction of DNA and histone methylation [12, 13], and modulation of the immune microenvironment [14]. This study aimed to develop early diagnostic methods and investigate the underlying pathogenesis of liver injury in BA using metabolomics.

## Materials and methods

### Ethics approval

Overall, 46 patients with BA and 22 controls were enrolled. Samples from choledochal cysts (*n* = 14), hepatoblastoma (HB, *n* = 3), diaphragmatic hernias (*n* = 2), and atrial septal (*n* = 3) were used as controls. All plasma samples were obtained before surgery (Table S1). Liver specimens were obtained from patients with BA and normal adjacent non-tumor tissues from patients with choledochal cysts and HB undergoing surgery. Written informed consent was obtained from the legal guardians. The protocol for using human participants was approved by the Faculty of Medicine’s Ethics Committee of Xin Hua Hospital (XHEC-D-2022-028). All methods were performed following the tenets of the Declaration of Helsinki. C57BL/6 male mice (approximately 6 weeks old) were used. Experiments with mice were approved by the Shanghai Jiao Tong University School of Medicine, affiliated with the Xin Hua Hospital Animal Care and Use Committee (XHEC-F-2021-074). All methods were performed following the relevant guidelines. The main reagents used are listed in Table S2.

### Immunohistochemistry analysis

Immunohistochemistry (IHC) was performed on the liver of paraffin sections from BA (*n* = 6), and controls (HB, *n* = 3; CC, *n* = 3) using the previously described diaminobenzidine chromogen method [15]. Paraffin-embedded tissues were deparaffinized using xylene and descending concentrations of ethanol. Next, 3% H_2_O_2_ was used to block the endogenous peroxidases. After antigen retrieval with citrate buffer, blocking was performed using 5% bovine serum albumin. The primary antibodies used are listed in Table S3. The slides were rinsed in phosphate-buffered saline (PBS) and incubated with the secondary antibody (Servicebio, Wuhan, China) for 1 h. Antibody binding was visualized using the liquid DAB substrate chromogen system (Dako, Glostrup, Denmark). The slides were rinsed in PBS and counterstained with hematoxylin. Case Viewer was used for IHC image analysis. Six liver samples were randomly selected from the BA and control groups for immunohistochemical staining, and five fields of view were randomly taken from each slide. The images were then quantized.

### Immunofluorescence (IF)

All the zebrafish were immediately fixed in 4% paraformaldehyde for 24 h and subjected to dehydration, clearing, and paraffin embedding. Sections were mounted on positively-charged slides after cutting at 4 μm thick, baked at 65 °C for 1 h, and stored at room temperature (RT) for later use. The slides were incubated with xylol and descending concentrations of ethanol. Endogenous peroxidases were blocked using 3% H_2_O_2_ for 10 min at RT. After antigen retrieval, blocking was performed with 5% bovine serum albumin for 30 min at RT. The antibodies were applied overnight at their optimal concentrations in a wet chamber at 4 °C. The slides were rinsed in PBS and incubated with the appropriate secondary antibody for 1 h at RT. Antibody binding was visualized using a liquid diaminobenzidine chromogen substrate system. Subsequently, the slides were rinsed with PBS and counterstained with hematoxylin. The primary antibodies used in this study are listed in Supplementary Table S3.

### D-2-HG in plasma and liver

D-2-HG concentrations in plasma were evaluated using a D-2-HG assay kit as previously described [16]. The assays were performed following the manufacturer’s instructions. Plasma samples and liver homogenates were added to a 96-well plate and mixed with 5 µL of 1 mM D-2-HG. A total of 50 μL reaction mix containing D-2-HG assay buffer, enzyme, and substrate mix were added into each well. The plates were incubated for 60 min at 37 °C and measured at 450 nm using a Synergy H1 Hybrid reader. The plasma samples of BA (*n* = 46) and controls (CC, *n* = 14, atrial septal defect, *n* = 3, and diaphragmatic hernias, *n* = 2) were used to measure D-2-HG levels in the blood. The D-2-HG levels in livers were determined in samples of BA (*n* = 13) and controls (CC, *n* = 8).

### Activities of TET enzymes in nuclear extracts

The liver tissues from BA (*n* = 19) and controls (CC, *n* = 6; HB, *n* = 2) were used to determine 5mC-Hydroxylase TET activity. Nuclear lysates were extracted using a Nuclear and Cytoplasmic Protein Extraction Kit, following the manufacturer’s instructions. Nuclear lysate concentrations were determined using the Enhanced Bicinchoninic Acid (BCA) Protein Assay Kit. TET activities were quantified in the nuclear fraction (7.5 μg protein) using Epigenase 5mC-Hydroxylase TET Activity/Inhibition Assay Kits via the detection of TET-converted hydroxymethylated products. The nuclear lysate incubation time was 90 min. The results were measured at 530 ex/590 em nm with a Synergy H1 Hybrid reader.

### Dissociation of the biliary tree

Tissue dissociation was performed as previously described [17]. Hank’s balanced salt solution without Ca^2+^ and Mg^2+^ was infused through the portal vein at 25–30 mL/min, and the inferior vena cava was cut open to drain the buffer. Hank’s balanced salt solution with Ca^2+^, Mg^2+^, and 0.05% collagenase II was then perfused into the livers. The biliary tree was cut into pieces, suspended in a 10 mL digestion medium (2 mg/mL collagenase IV, 0.01 mg/mL DNase I), and shaken at 37 °C for 30 min. The digested tissues were filtered using a 40 μm cell strainer and centrifuged at 800*g* for 10 min at 4 °C.

### Magnetic-associated cell sorting

Hepatic progenitor cells were isolated by magnetic associated cell sorting using CD326 (EpCAM) MicroBeads following the manufacturer’s protocol. Total disassociated cells were suspended in a running buffer and incubated with 10 µL CD326 (EpCAM) MicroBeads for 15 min at 4 °C. The mixture was sorted using an LS column. The column was then rinsed with a running buffer to elute the cells. The cells were counted and centrifuged at 450×*g* for 5 min for follow-up experiments.

### Organoid culture medium

EpCAM ^+^ cells (1 × 10^5^) were first resuspended in 15 µL ice-cold serum-free medium and mixed with 40 µL Matrigel on ice. Approximately, 50 µL of the cell suspension was seeded in a prewarmed (37 °C) 24-well plate. The mixture was added to the bottom of the well and incubated at 37 °C for 5–10 min. The plates were then incubated upside-down at 37 °C for 5 min to allow for the even distribution of cells. After the Matrigel solidified, 500 µL of the medium was added to each well. Organoid culture medium was a 1:1 mixture of advanced Dulbecco’s Modified Eagle Medium/Nutrient Mixture F-12 (DMEM/F12) and conditioned medium from L-WRN cells [18], supplemented with 25 mM HEPES, penicillin/streptomycin, 1% N2, 1% B27, 50 ng/ml epidermal growth factor, and 100 ng/ml fibroblast growth factor 10. The rho-associated coiled kinase inhibitor Y-27632 (10 µM, Tocris) was added during the first 3 days of culture. The medium was changed once every 2 days. Primary organoids at passages 0 and 1 were used for subsequent experiments.

### Passaging and freezing of organoids

Organoids usually pass 7–9 days when they start to collapse. The matrix and organoids were pipetted up and down for passaging into small pieces in 500 µL ice-cold advanced DMEM/F12 medium. The mixture was then transferred to 15 mL tubes, 10 mL of Advanced DMEM/F12 medium was added, and the tubes were placed on ice for 10–15 min to dissolve the Matrigel. The tubes were centrifuged at 4 °C and 300×*g* for 10 min. The supernatant was discarded, and the cell pellets were resuspended in 50–60 µL of Matrigel. Approximately, 500 µL of prewarmed organoid culture medium was added after solidification of the matrigel. The medium was changed every 3 days.

### Organoids exposure to D-2-HG

Matrigel and organoids were dissolved in ice-cold PBS and centrifuged. Organoids were digested into single cells using trypsin. The cells were counted following the addition of 200 µL organoid medium. After dilution, approximately 1000 cells were divided into different tubes and centrifuged (300 g, 5 min) at 4 °C. Four replicates were analyzed for the control (PBS alone) or D-2-HG treatments. Five hundred microliters of organoid medium containing PBS for the control or D-2-HG (1, 2, 5, or 10 mM) for each treatment were prepared. The cell pellets were incubated (10 min) with 10 µL of the prepared medium containing the appropriate compounds. Matrigel (50–60 µL) was added, and the rest of the corresponding medium with compounds (500 µL) was added per well after the matrigel solidification. The medium was then changed to the corresponding D-2-HG concentration. Images of all organoids were taken under the same magnification on days 0, 3, and 6, and organoid growth was determined by the organoid size. Image J was used to measure and calculate the diameter of the organoids.

### Western blotting

For clinical samples, the Western blotting was performed on the liver tissues of BA (*n* = 4) and CC (*n* = 4). For mice livers, EpCAM^+^ cells were isolated from the liver of mice and treated with D-2-HG (5 mM) for 4 h. Approximately, 1 × 10^5^ EpCAM^+^ cells were homogenized in 200 μL of radioimmunoprecipitation assay lysis buffer (Servicebio Inc, Wuhan, China) supplemented with a protease inhibitor cocktail. Bicinchoninic Acid reagent (Beyotime Inc., Shanghai, China) was used to determine protein concentration. Equal amounts of protein were separated on NuPAGE 10% Bis–Tris gels (Invitrogen, Carlsbad, CA) and transferred onto polyvinylidene difluoride membranes. After blocking in 5% nonfat milk at RT for 1 h, membranes were incubated with the primary antibodies overnight at 4 °C. Subsequently, the membranes were washed three times for 30 min with Tris Buffered saline with Tween (containing 0.1% Tween-20), then incubated with secondary antibodies. After washing with Tris Buffered Saline with Tween, signals were detected using an enhanced chemiluminescence reagent kit. The primary antibodies used are listed in Supplementary Table S3.

### Quantitative real-time polymerase chain reaction

Total RNA from organoids was extracted using an RNAprep Pure Micro Kit. Cold PBS, (500 µL) was added to the ice for mouse liver organoids to dissolve Matrigel. It was carefully pipetted several times to resuscitate organoids in the well. The suspension was transferred to a 1.5-mL tube and centrifuged at 600*g* for 5 min at 4 °C. Total RNA was extracted following the manufacturer’s instructions. RNA concentration was determined using nanocrystal spectroscopy. The Hifair® II 1st Strand cDNA Synthesis SuperMix was used for reverse transcription. The ViiA 7 Real-Time PCR system and Hieff® qPCR SYBR Green Master Mix were used for real-time PCR. PCR reaction was incubated at 95 °C for 10 min, 95 °C for 15 s, and 60 °C for 1 min in 384-well plates, with 40 repeated cycles. For human liver specimens, a total RNA was extracted from BA (*n* = 45) and controls (14 CC and 3 normal adjacent non-tumor tissues from patients with HB). All sample analyses were repeated thrice, and data were normalized to the endogenous control glyceraldehyde-3-phosphate dehydrogenase (GAPDH). The relative RNA expression was calculated using the ^ΔΔ^Ct method. The primers used are listed in Table S4.

### OCR measurement

Cells (2 × 10^4^) from organoids were plated in an XF96 cell culture microplate, coated with a 1:10 dilution of Matrigel in PBS, and allowed to settle at RT for 1 h. Organoids were evenly seeded in each well with an organoid culture medium. The following day, the growth medium was changed to a bicarbonate-free assay medium with PBS or D-2-HG and incubated at 37 °C for 1 h in a CO_2_-free incubator. Organoids were run on an XF96e Analyzer for a Mito Stress Test in XF DMEM medium (pH 7.4) with 25 mM glucose, 2 mM pyruvate, and 2 mM glutamine following the manufacturer’s protocol and using the standard drug concentrations of 1 µM oligomycin, 1.2 µM carbonyl cyanide-4-(trifluoromethoxy) phenylhydrazone, 0.5 µM rotenone, and 0.5 µM antimycin A.

### ECAR measurement

Cells (2 × 10^4^) from organoids were plated in an XF96 cell culture microplate, coated with a 1:10 dilution of Matrigel in PBS, and allowed to settle at RT for 1 h. Organoids were evenly seeded in each well with an organoid culture medium. The following day, the growth medium was changed to a bicarbonate-free assay medium with PBS or D-2-HG and incubated at 37 °C for 1 h in a CO_2_-free incubator. The ECAR was measured using an XF96 Extracellular Flux Analyzer under basal conditions and following the addition of 10 mM glucose, 1 μM oligomycin, and 50 mM glucose analog—2-deoxyglucose following the manufacturer’s protocol.

### Zebrafish bioassay and analyses

The wild-type strains were raised following the protocols approved by the Shanghai Jiao Tong University School of Medicine affiliated with Xin Hua Hospital Animal Care and Use Committee. Zebrafish larvae (5 dpf) were exposed to D-2-HG dissolved in embryo medium at 0–20 mM (0, 0.5, 1, 5, 10, or 20 mM). Fifteen larvae were placed in each well of a 24-well plate for 16 h. The medium containing each compound was changed every 12 h. All tests were repeated thrice, with at least 30 treated larvae and the same number of control larvae. After treatment, the larvae were fixed with 4% paraformaldehyde and treated with annexin A4 or HNF-4α for IF staining. The relative average fluorescence intensity was analyzed using the Image J software.

### Statistical analyses

All experiments were repeated at least twice with identical or similar results. GraphPad Prism8.0 software was used for data statistics and Spearman’s correlation analysis. Data represented biological replicates. A two-sided analysis was used in this study. The mean ± SD was plotted in all the figures. The comparison between the two groups was analyzed using Student’s *t*-test. Statistical significance was determined using ANOVA with Bonferroni correction for data from three or more groups. Statistical significance was set at *P* < 0.05.

## Results

### D-2-HG is increased in BA plasma and correlated with liver injury

**Fig. 1:**
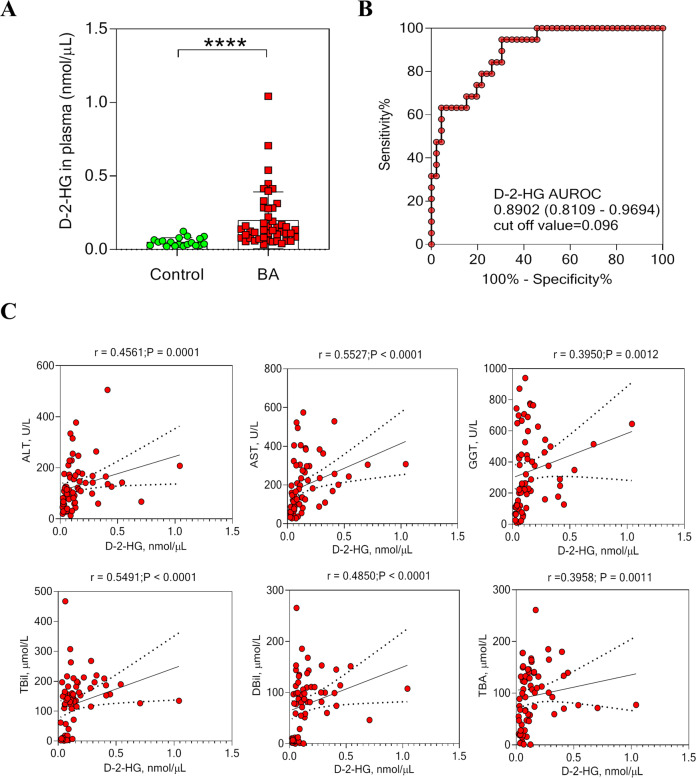
D-2-HG contents were increased in BA patients and correlated with liver injury. **A** The D-2-HG expression was detected in the plasma of BA patients (*n* = 46) and controls (*n* = 19). **B** The receiver operating characteristic (ROC) was constructed by plasma D-2-HG. **C** Correlation analysis of plasma D-2-HG levels with alanine aminotransferase (ALT), aspartate aminotransferase (AST), γ-glutamyltransferase (GGT), total bilirubin (TBil), direct bilirubin (DBil), and total bile acid (TBA) levels. Statistical significance: ****p < 0.0001.

We recently used ultra-performance liquid chromatography-triple quadrupole mass spectrometry to profile metabolomics in dried blood spots of newborns [8]. It was shown that 2-HG increased significantly in the DBS of patients with BA compared with the controls. Consistently, we showed that plasma D-2-HG levels were significantly elevated in children with BA (*n* = 46) compared with matched controls (*n* = 19) (Fig. 1A). The area under the receiver operating characteristic curve of plasma D-2-HG was 0.8902 (95% confidence interval [CI]: 0.8109‒0.9694; Fig. 1B), and the cutoff value of the ROC was 0.096 nmol/μL. As shown in Fig. 1C, plasma D-2-HG levels were positively correlated with the plasma markers of liver injury and cholestasis. D-2-HG correlated with alanine aminotransferase (r = 0.4561, *P* = 0.0001), aspartate aminotransferase (*r* = 0.5527, *P* < 0.0001), γ-glutamyltransferase (*r* = 0.395, *P* = 0.0012), total bilirubin (*r* = 0.5491, *P* < 0.0001), direct bilirubin (*r* = 0.485, *P* < 0.0001), and total bile acid (*r* = 0.3958, *P* = 0.0011) levels (Fig. 1C).

### D-2-HG is accumulated in the liver of patients with BA and alters the microenvironment

**Fig. 2:**
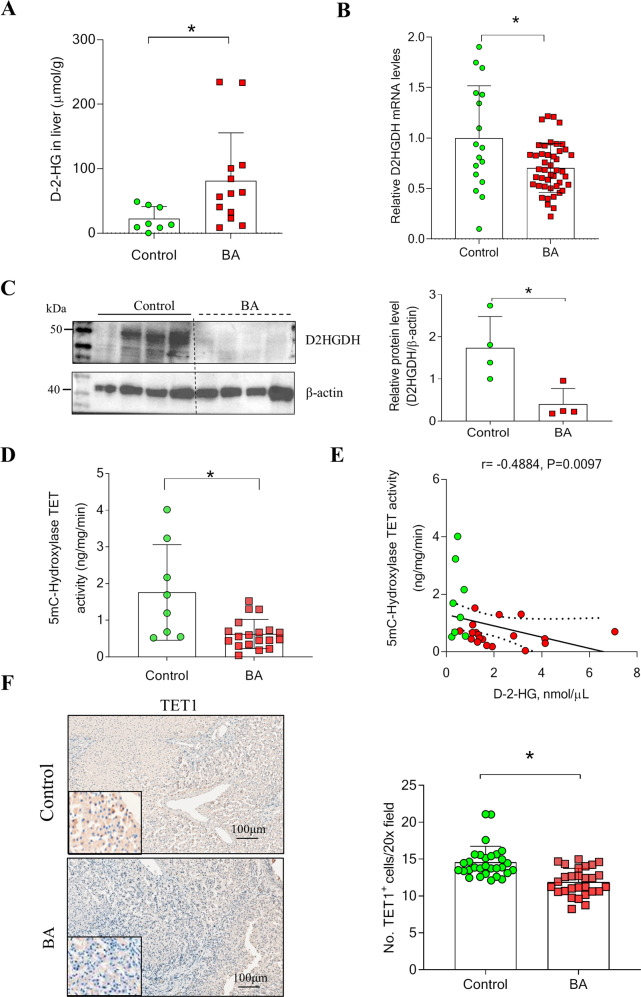
Accumulation of D-2-HG was correlated with TET activity in BA patients. **A** The D-2-HG expression was detected in the livers of BA (*n* = 13) and controls (*n* = 8). **B** The relative expression of D2HGDH mRNA in the livers of BA (*n* = 45) and controls (*n* = 17). **C** Representative images of western blotting (WB) for D2HGDH. Quantification of D2HGDH against β-actin. **D** 5mC-Hydroxylase TET activity was determined in the livers of BA patients (*n* = 19) and controls (*n* = 8). **E** Correlation analysis of plasma D-2-HG levels with 5mC-Hydroxylase TET activity in liver tissues. **F** Representative Immunohistochemistry (IHC) images of TET1 in livers of BA patients (*n* = 6) and controls (*n* = 6). The quantification of TET1 positive cells. Statistical significance: *p < 0.05.

We measured D-2-HG levels in the liver tissues of patients with BA and their matched controls. Liver D-2-HG levels in the BA group (*n* = 13) were significantly higher than those in the control group (*n* = 8) (Fig. 2A). Under normal conditions, D-2-HG dehydrogenase is responsible for the breakdown of D-2-HG [19, 20]. In response to hypoxia, hypoxia-inducible factor-1alpha is important for generating D-2-HG from 5-carbon-ketoglutarate [21]. Quantitative real-time polymerase chain reaction and western blotting demonstrated that D-2-HG dehydrogenase (D2HGDH) mRNAs and protein expression were decreased in patients with BA (Fig. 2B, C). IHC images also showed that the hypoxia-inducible factor-1alpha protein was increasingly expressed in the liver of patients with BA compared with controls (Fig. S1). Studies have reported that the accumulation of D-2-HG in the nucleus inhibits the 10–11 translocation (TET) family of 5mC hydroxylases, which play a role in DNA demethylation [13, 22, 23]. Several studies have indicated a gradual loss of 5mC-hydroxylase in several liver pathologies, such as liver cancer [24–27]. Here, we found a significant decrease in 5mC-hydroxylase TET activity in the liver of patients with BA (*n* = 19) compared with that in the controls (*n* = 8) (Control vs. BA, 1.756 ± 1.304 vs. 0.6252 ± 0.3984 ng/mg/min, *P* < 0.05; Fig. 2D). Correlation analysis showed that plasma D-2-HG levels were negatively correlated with 5mC-Hydroxylase TET activity (r = − 0.4884, *P* = 0.0097; Fig. 2E). IHC analysis also showed that TET1 protein expression was reduced in the liver of patients with BA compared with the controls (Fig. 2F).

### Liver regeneration is impaired in patients with BA

**Fig. 3:**
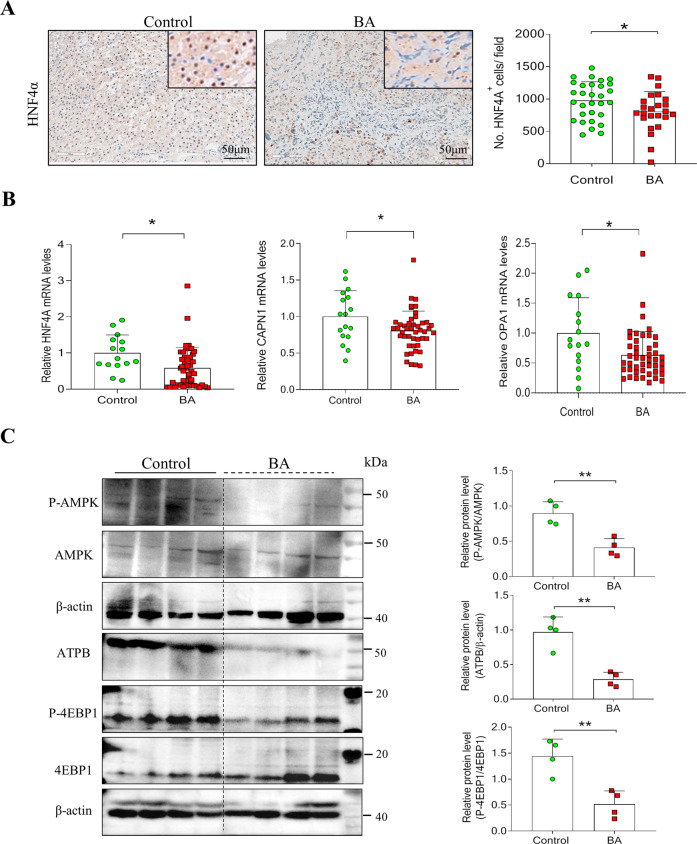
Mitochondrial respiration and ATP synthase decreased in BA patients. **A** Representative immunohistochemistry (IHC) images of HNF-4α in livers of BA patients (*n* = 6) and controls (*n* = 6). The quantification of HNF-4α^+^ cells. **B** The relative expression of HNF4A, CAPN1, and OPA1 mRNAs in the liver of BA and controls. **C** Representative images of western blotting (WB) for P-AMPK, AMPK, ATPB, P-4EBP1, and 4EBP1 and quantification of WB. Statistical significance: *p < 0.05.

Hepatocyte nuclear factor-alpha (HNF-4α) is a master regulator of the hepatocyte phenotype [28]. Lipid nanoparticle-mediated HNF-4α gene (HNF4A) ameliorates fibrosis and cirrhosis. It also improves functions in mice and humans [29]. We showed that the number of HNF-4α positive cells was reduced in the liver tissues of patients with BA compared with that in the controls (Fig. 3A). Consistently, HNF4A mRNAs decreased in patients with BA (Fig. 3B). In addition, calpain 1 (CAPN1) and optic atrophy 1 (OPA1) genes, closely related to mitochondrial energy metabolism, decreased significantly in the liver of patients with BA (Fig. 3B). Consistently, ATPase beta-chain protein, an ATP synthase, was significantly reduced in the liver of patients with BA compared with the controls (Fig. 3C). Previous studies have identified AMP-activated protein kinase (AMPK) as an upstream mediator of OPA1 [30]. Western blotting demonstrated that the phosphorylation of AMPK and eIF4E binding protein-1 was suppressed in the liver of patients with BA (Fig. 3C). The expression of the mammalian target of rapamycin (mTOR) and cyclin D1 was also decreased in the liver of patients with BA compared with the controls (Figure S2).

### D-2-HG suppresses the growth of liver organoids

**Fig. 4:**
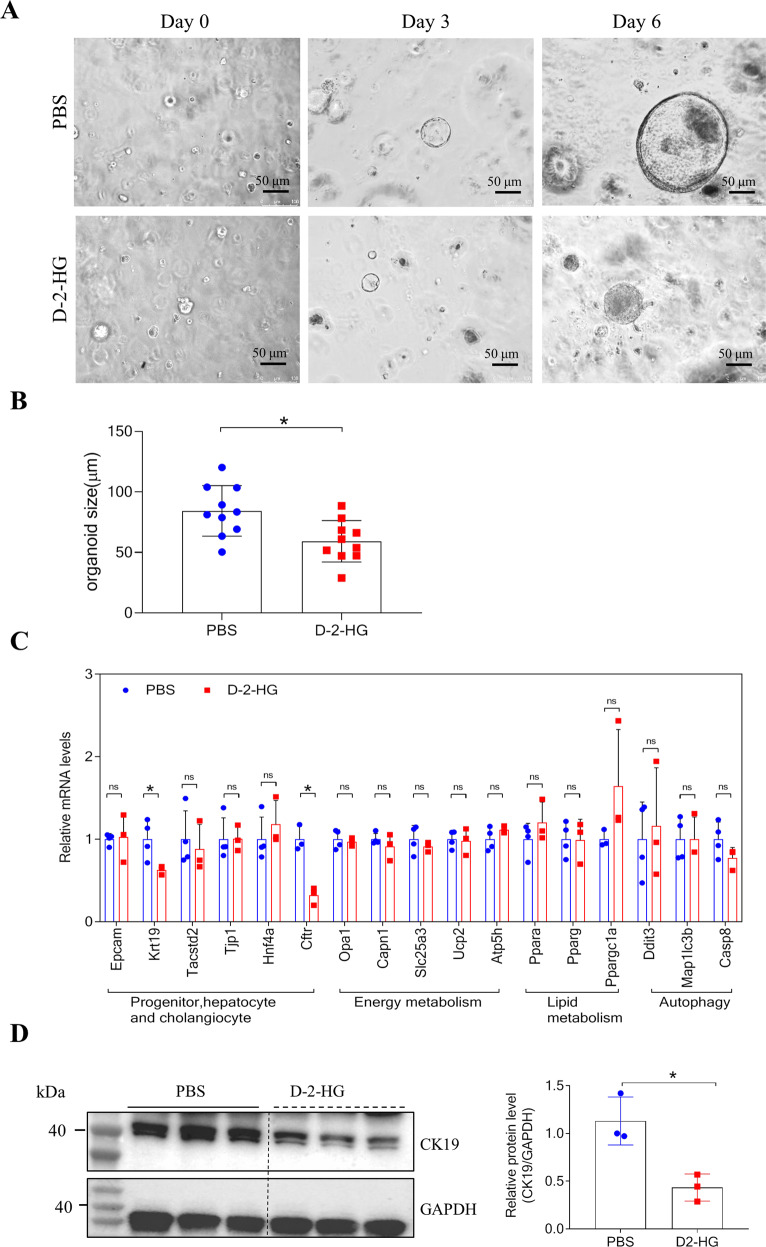
D-2-HG restrained the growth of liver organoids. **A** Representative size and morphology of culture organoids treated with PBS, or 5 mM D-2-HG at the indicated time. **B** Quantification for the size of organoids in panel (**A**). **C** The relative expression of involved genes mRNA in the liver organoids with or without 5 mM D-2-HG exposure. **D** At 4 h after PBS or 5 mM D-2-HG exposure, the proteins of the EpCAM+ cells were extracted for western blotting (WB). Representative images of WB for CK19. Quantification of CK19 against GAPDH. ns, not significant, *P < 0.05.

Given the importance of liver progenitors for liver regeneration, we sorted Epcam^+^ bipotent hepatoblast progenitors and established liver organoids under previously defined organoid culture conditions [31]. Epcam^+^ cells were embedded in Matrigel containing the organoid culture factors epidermal growth factor, fibroblast growth factor 10, and medium from L-WRN cells [32]. Two-dimensional perimeter tracing indicated that a co-culture with D-2-HG (5 mM) significantly decreased organoid size at the indicated times (Fig. 4A, B). Quantitative real-time polymerase chain reaction analysis revealed that D-2-HG did not alter the liver progenitor cell surface markers Epcam or Tacstd2 in organoids (Fig. 4C). Moreover, D-2-HG significantly inhibited the mRNA expression of the cholangiocyte marker Krt19 and cystic fibrosis transmembrane conductance regulator (Fig. 4C). Consistently, D-2-HG significantly suppressed the expression of cytokeratin 19 protein in liver progenitors (Fig. 4D).

### D-2-HG impairs mitochondrial respiration in liver organoids

**Fig. 5:**
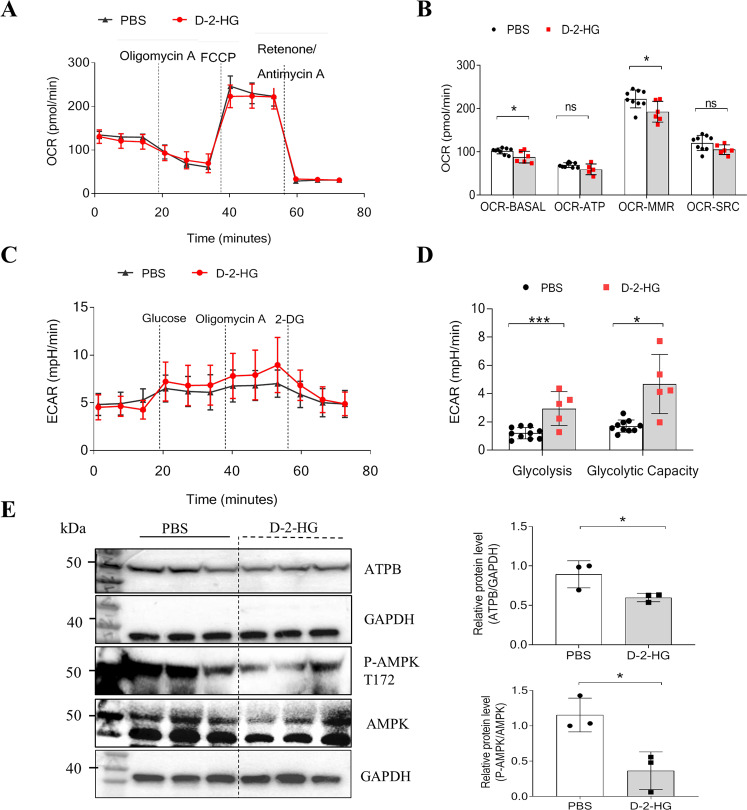
D-2-HG inhibited mitochondrial respiration in liver organoids. **A** Representative OCR experiments were analyzed in liver organoids treated with PBS, or 5 mM D-2-HG, using the Seahorse XF96 extracellular flux analyzer. Each group, *n* = 6–10. **B** Levels of mitochondrial respiratory function: basal respiration (OCR-BASAL), ATP production (OCR-ATP), maximal respiration (OCR-MMR), and spare respiratory capacity (OCR-SRC) in liver organoids. **C** Representative ECAR experiments performed on liver organoids treated with PBS or 5 mM D-2-HG. **D** Levels of glycolysis and maximal glycolytic capacity in liver organoids. **E** At 4 h after PBS or 5 mM D-2-HG exposure, the proteins of the EpCAM+ cells were extracted for western blotting (WB). The WB was used to determine the amounts of P-AMPK, AMPK, and ATPB (*n* = 3). ns, not significant, *P < 0.05, ***P < 0.001.

Using a Seahorse XF96 Analyzer, we examined the effects of D-2-HG on the oxygen consumption rate (OCR) in liver organoids. The OCR is an indicator of mitochondrial respiratory capacity and energy production. Inhibitors, such as oligomycin A, carbonyl cyanide-4-(trifluoromethoxy)phenylhydrazone, and rotenone/antimycin A, were used to measure oxygen consumption during different mitochondrial respiratory processes (Fig. 5A). As shown in Fig. 5B, D-2-HG-treated organoids had significantly lower basal mitochondrial respiration (OCR-BASAL) (87.57 ± 13.42 pmol/min, *P* < 0.05) compared with the control (101.84 ± 6.66 pmol/min) (Fig. 5B). Oxygen consumption linked to mitochondrial ATP production (OCR-ATP) can be determined by adding the ATP synthase inhibitor oligomycin A. The amount of OCR-ATP production was suppressed by D-2-HG; however, it could not attain significance (Fig. 5B). The maximal ATP mitochondria output can be determined by adding carbonyl cyanide-4-(trifluoromethoxy)phenylhydrazone, which disrupts mitochondrial membrane potential. Maximal respiration (OCR-MMR) was significantly lower in D-2-HG-treated organoids (192.33 ± 23.94 pmol/min, *P* < 0.05) compared with the control (221.99 ± 20.69 pmol/min) (Fig. 5B). The spare respiratory capacity, an indicator of cells’ ability to respond to increased energy demand, was also decreased in D-2-HG-treated organoids (Fig. 5B). We also measured the extracellular acidification rate (ECAR) in organoid cultures. Glucose (10 mM) was added to increase the ECAR, which served as the rate of glycolysis under basal conditions (Fig. 5C, D). It showed a significantly increased rate of glycolysis in D-2-HG (2.94 ± 1.20 mpH/min, *P* < 0.001) organoids compared with PBS control (1.20 ± 0.39 mpH/min) (Fig. 5C, D). The subsequent addition of oligomycin A shifts energy production to glycolysis and increases ECAR, serving as the maximum glycolytic capacity. We revealed a significantly increased maximum glycolytic capacity in D-2-HG (4.68 ± 0.43 mpH/min, *P* < 0.001) organoids compared with control (1.70 ± 0.43 mpH/min) (Fig. 5C, D). We also found that the expression of ATPase beta-chain protein and the phosphorylation of AMPK, a cellular energy sensor, was reduced by D-2-HG administration in liver progenitors (Fig. 5E).

### D-2-HG inhibits mTOR signaling and protein kinase B (AKT) activation

**Fig. 6:**
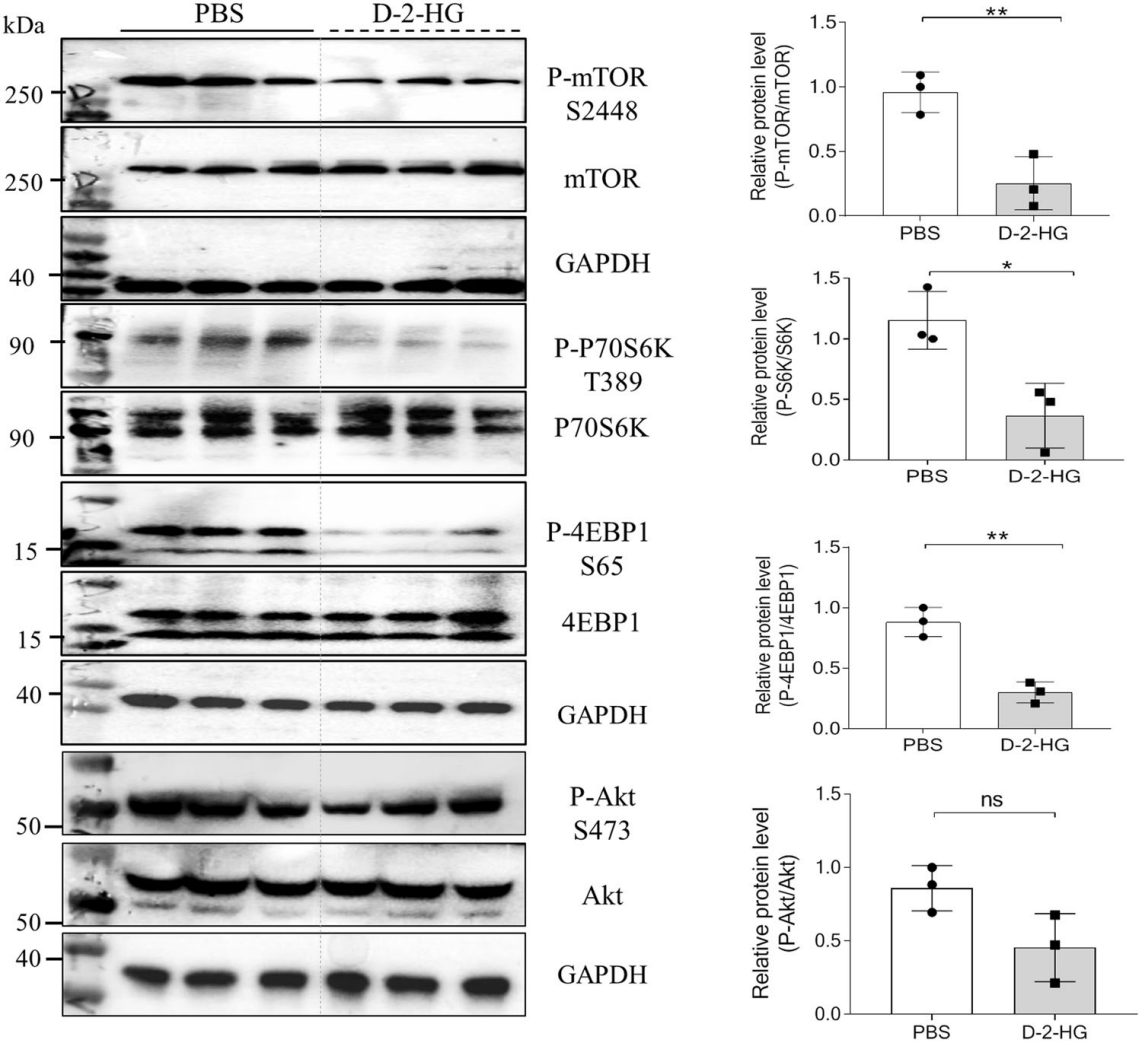
D-2-HG suppressed the mTOR signaling. At 4 h after PBS or 5 mM D-2-HG exposure, the proteins of the EpCAM+ cells were extracted for western blotting (WB). Representative WB analysis for the key proteins in the mTOR signaling, including mTOR, p70 S6K, 4EBP1, and AKT. Quantification for the expression of key proteins in the mTOR signaling. ns, not significant, *P < 0.05, **P < 0.01.

Since the mammalian target of rapamycin (mTOR) signaling plays an essential role in liver growth [33], we evaluated whether D-2-HG-mediated effects in liver organoids depended on mTOR signaling activation. As shown in Fig. 6, mTOR phosphorylation at Ser 2448 of mTOR was reduced after 4 h of D-2-HG exposure (Fig. 6). This subsequently caused a decrease in the phosphorylation of eIF4E binding protein-1 (4EBP1) and the activation of p70 ribosomal S6 protein kinase (P70 S6K), both of which are essential steps in the stimulation of protein translation (Fig. 6). In addition, D-2-HG reduced the phosphorylation of protein kinase B (AKT, Ser 473) (Fig. 6).

### D-2-HG exposure caused liver degeneration in zebrafish larvae

**Fig. 7:**
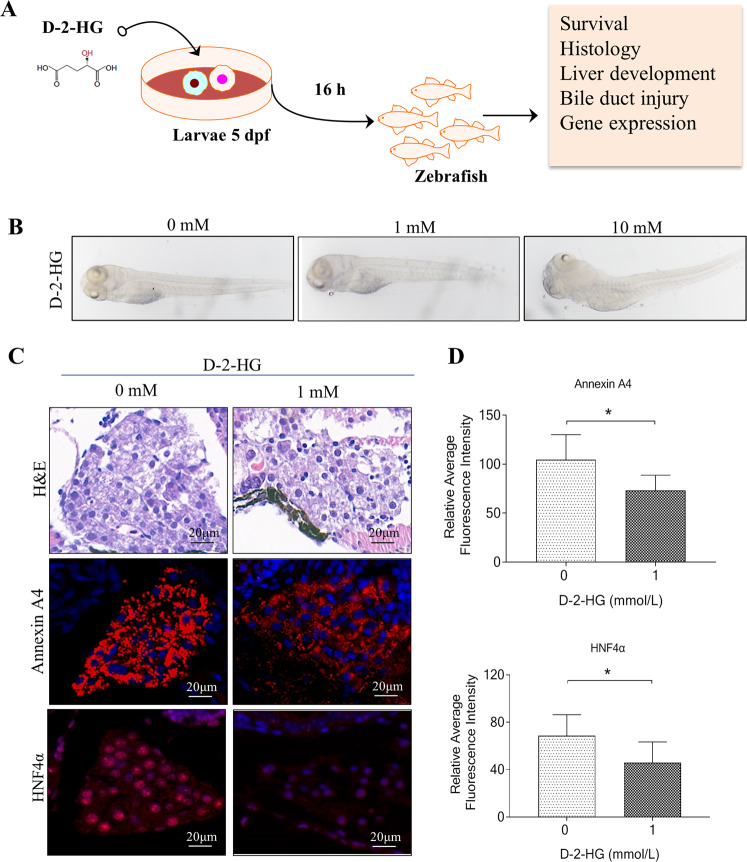
D-2-HG exposure caused liver degeneration in zebrafish. **A** Experimental scheme depicting the treatment of 5 dpf larvae with different concentrations of D-2-HG (0, 0.5, 1, 5, 10, and 20 mM) for 16 h before analysis. **B** The whole morphology of larvae. **C** Livers of 5-dpf larvae were performed with hematoxylin-eosin (H&E) staining and immunostained with anti-Annexin A4 and HNF-4α fluorescence intensity. **D** Quantification of Annexin A4 and HNF-4α in the panel (**C**). *P < 0.05, **P < 0.01.

Given that mTOR signaling plays an essential role in biliary epithelial cell-driven liver regeneration in mice and zebrafish [33–35], we investigated whether D-2-HG-mediated mTOR signaling inactivation impaired liver development or regeneration in a zebrafish model. The larvae at 5 days postfertilization (dpf) were incubated with D-2-HG at different concentrations (0, 0.5, 1, 10, or 20 mM) for 16 h (Fig. 7A). Over 70% of the larvae died after treatment with high-dose D-2-HG (10‒20 mM), whereas low-dose D-2-HG (0.5‒1 mM) only had minor effects on larvae survival (Fig. 7B, C). As expected, high-dose D-2-HG induced apparent changes in the liver, causing biliary and hepatic injuries or degeneration (Fig. 7B). Annexin A4 has been identified as a hepatopancreas factor involved in liver cell survival, which autonomously requires cells for survival in developing zebrafish liver [36]. Larvae immunostained with anti-annexin A4 showed that D-2-HG significantly reduced annexin A4 expression relative to that of untreated cells (Fig. 7C, D). Additionally, we showed that HNF-4α was significantly reduced in the liver of larvae following D-2-HG treatment (Fig. 7C, D).

## Discussion

**Fig. 8:**
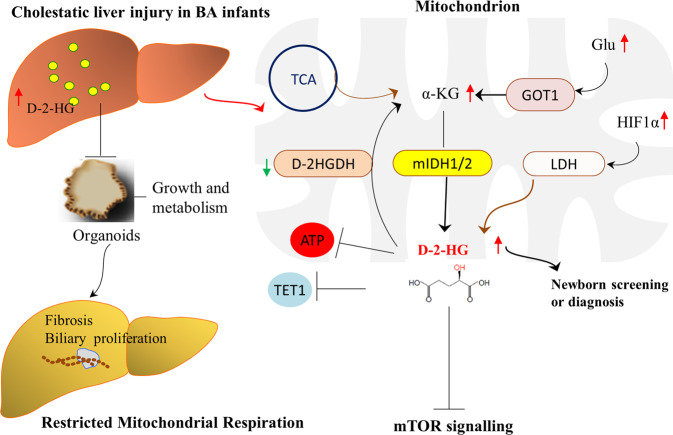
Schematic diagram of potential mechanisms that D-2-HG involved in liver regeneration in BA patients. Biliary atresia is incurable and needs liver transplantation. Using targeted metabolomics, we unexpectedly identified a metabolite 2-hydroxyglutarate (2-HG) that has great potential in diagnosis or newborn screening. By using ‘mini organs of liver’ in vitro and a zebrafish model in vivo, we demonstrate D-2-HG accumulates in the liver and alters the hepatic microenvironment, and subsequently impairs liver regeneration via reducing the mitochondrial respiration and ATP synthase. Our findings reveal D-2-HG has a novel pathogenic mechanism that could have important diagnostic and therapeutic implications.

In our previous study, we evaluated the metabolic profile of DBS from newborns, which demonstrated the potential of 2-HG for birth screening in infants with BA. The present study showed that plasma and liver D-2-HG expression were significantly upregulated in patients with BA. Furthermore, plasma and liver D-2-HG levels were closely correlated with the severity of the cholestatic liver injury and liver repair retardation in patients with BA. In addition, we identified a well-reported D-2-HG regulatory mechanism involved in liver regeneration. Our study suggested that accumulated D-2-HG altered the metabolic microenvironment and impaired liver regeneration by regulating mitochondrial respiration, ATP synthase, and mTOR signaling (Fig. 8).

Early diagnosis of BA is associated with the age of patients in the KPE operation and its outcomes. In this study, we suggest that increased 2-HG levels have the potential to screen or diagnose infants with BA. 2-HG is a metabolite strictly regulated by several enzymes [10, 37]. We suggest that increased D-2-HG levels in the plasma and liver of patients with BA may be attributed to the dysregulation of D-2-HG dehydrogenase. The levels of ATP synthesis and energy metabolism in the liver decreased in patients with BA, which was related to liver damage. Therefore, plasma D-2-HG was positively correlated with liver injury in patients with BA. D-2-HG is a known 5-carbon-ketoglutarate antagonist that inhibits histone demethylases and the TET family of DNA dioxygenases [12, 38]. TET1 is involved in DNA demethylation by modulating 5-hydroxymethycytosine formation [39], which plays a vital role in the development and function of the human liver [27]. In addition, TET1 catalytic activity is required for cholangiocyte organoid formation and liver regeneration [40]. We found that the activity of the TET family of 5mC hydroxylases decreased in the liver of patients with BA and was negatively correlated with D-2-HG levels. Moreover, TET1 expression was reduced in the liver tissues of infants with BA. Therefore, altered D-2-HG may have contributed to liver damage partly via epigenetic modulation of TET activity.

Following a severe or chronic liver injury, cholangiocytes act as liver stem cells during impaired hepatocyte regeneration [41]. We showed that Epcam^+^ cells clonally expand as self-renewing liver organoids with the capacity to differentiate into hepatocytes and biliary cells. D-2-HG administration significantly inhibited liver organoid growth and differentiation. Additionally, D-2-HG-treated liver progenitors showed increased aerobic glycolysis and reduced mitochondrial respiration and ATP production. ATPase beta-chain protein and the phosphorylation of the energy sensor AMPK were reduced in D-2-HG treated-liver progenitors. mTOR is an important energy/nutrient sensor that regulates energy production, protein synthesis, and autophagy to maintain metabolic homeostasis [42]. Furthermore, mTOR is essential for liver regeneration [33, 43–45]. From the above discussion, we concluded that D-2-HG inhibited mTOR signaling, which is consistent with previous studies [46]. Moreover, AKT phosphorylation was inhibited by D-2-HG treatment. mTOR is a direct AKT target by regulating the phosphorylation of mTOR Ser 2448 in response to growth factors and amino acids [47]. Therefore, we proposed that D-2-HG inhibited mTOR signaling, possibly by modulating AKT activity.

One important future direction of potential clinical application is using D-2-HG to assist in early BA diagnosis or to reduce the effect of D-2-HG on energy metabolism to improve liver regeneration in children with BA. However, this study had some limitations. For example, it is unclear whether D-2-HG affects the regeneration of other organs. Interactions between different organs may affect 2-HG metabolism.

In conclusion, we propose that D-2-HG accumulates in the liver of patients with BA and induces metabolic alterations in the microenvironment that impair liver repair. D-2-HG inhibits liver regeneration by reducing mitochondrial respiration, increasing aerobic glycolysis, and inhibiting mTOR signaling.

## Supplementary information

Supporting Information

checklist-CDDIS-22-2395

## Data Availability

The data generated or analyzed during this study are available from the corresponding author upon reasonable request.
