# Supplementary material for: Metabolic regulation of cholestatic liver injury by D-2-hydroxyglutarate with the modulation of hepatic microenvironment and the mammalian target of rapamycin signaling
Source: Cell Death Dis. 2022 Nov 26;13(11):1001. doi: 10.1038/s41419-022-05450-z (PMC9701230; doi:10.1038/s41419-022-05450-z)
Supplement: Supplementary file 1 — Supporting Information [file 41419_2022_5450_MOESM1_ESM.docx]

**Metabolic Regulation of Cholestatic Liver Injury by D-2-Hydroxyglutarate with the Modulation of Hepatic Microenvironment and the Mammalian Target of Rapamycin Signaling**

**Running title:** D-2-HG, a diagnostic biomarker in infants with BA

[Xinbei Tian](https://www.ncbi.nlm.nih.gov/pubmed/?term=Tian%20X%5BAuthor%5D&cauthor=true&cauthor_uid=34168124)^#,1,2,4^,[Ying Wang](https://www.ncbi.nlm.nih.gov/pubmed/?term=Wang%20Y%5BAuthor%5D&cauthor=true&cauthor_uid=34168124)^#,1,2,4^, [Ying Lu](https://www.ncbi.nlm.nih.gov/pubmed/?term=Lu%20Y%5BAuthor%5D&cauthor=true&cauthor_uid=34168124)^2,3^, Bo Wu^1,2,4^,[Shanshan Chen](https://www.ncbi.nlm.nih.gov/pubmed/?term=Chen%20S%5BAuthor%5D&cauthor=true&cauthor_uid=34168124)^1,2,4^, [Jun Du](https://www.ncbi.nlm.nih.gov/pubmed/?term=Du%20J%5BAuthor%5D&cauthor=true&cauthor_uid=34168124)^2,3^, [Wei Cai](https://www.ncbi.nlm.nih.gov/pubmed/?term=Cai%20W%5BAuthor%5D&cauthor=true&cauthor_uid=34168124)^*,1,2,3,4^  and [Yongtao Xiao](https://www.ncbi.nlm.nih.gov/pubmed/?term=Xiao%20Y%5BAuthor%5D&cauthor=true&cauthor_uid=34168124)^*,1,2,3,4^

^1^ Division of Pediatric Gastroenterology and Nutrition, Xin Hua Hospital, School of Medicine, Shanghai Jiao Tong University, Shanghai, 200092, China

^2^ Department of Pediatric Surgery, Xin Hua Hospital, School of Medicine, Shanghai Jiao Tong University, Shanghai 200092, China.

^3^ Shanghai Institute for Pediatric Research, Shanghai 200092, China.

^4^ Shanghai Key Laboratory of Pediatric Gastroenterology and Nutrition, Shanghai 200092, China.

**Content of Supplementary Information**

Table S1. The information of patients with biliary atresia and control subjects, Page 2 - 4

Table S2. Reagents and Source, Page 5

Table S3. Antibody information, Page 6

Table S4. The primer sequences for qRT-PCR, Pages 7 - 8

Figure S1. Hepatic HIF1α increased in BA patients. Page 9

Figure S2. Impaired mTOR signaling in livers of BA patients. Page 10

Original and uncropped western-blot bands in this study, Pages 11-19

**Table S1. The information of patients with biliary atresia and control subjects**

| **Patient** | **Gender** | **Age (d)** | **Alanine aminotransferase (U/L)** | **Aspartate aminotransferase (U/L)** | **γ-glutamyltranspeptidase (U/L)** | **Total Bilirubin (μM)** | **Direct Bilirubin (μM)** |
| --- | --- | --- | --- | --- | --- | --- | --- |
|  |  |  |  |  |  |  |  |
| Biliary Atresia 1 | Female | 43 | 126 | 202 | 215 | 156.4 | 114 |
| Biliary Atresia 2 | Female | 11 | 115 | 278 | 230 | 143.5 | 69 |
| Biliary Atresia 3 | Male | 40 | 193.2 | 108.2 | 315 | 122 | 90 |
| Biliary Atresia 4 | Female | 65 | 172 | 252 | 1975 | 436.9 | 312.7 |
| Biliary Atresia 5 | Male | 29 | 40 | 85 | 348 | 467.4 | 265.5 |
| Biliary Atresia 6 | Male | 46 | 142 | 244 | 235 | 189.8 | 151.4 |
| Biliary Atresia 7 | Female | 61 | 162 | 209 | 2099 | 182.4 | 134.5 |
| Biliary Atresia 8 | Female | 72 | 109 | 115 | 263 | 110.3 | 58.9 |
| Biliary Atresia 9 | Male | 65 | 255 | 366 | 788 | 145.8 | 110.9 |
| Biliary Atresia 10 | Male | 56 | 132 | 208 | 1400 | 158.9 | 87.3 |
| Biliary Atresia 11 | Female | 72 | 255 | 305 | 434 | 263.2 | 185.5 |
| Biliary Atresia 12 | Female | 61 | 125 | 324 | 423 | 154 | 112.9 |
| Biliary Atresia 13 | Female | 25 | 40 | 78 | 645 | 157.4 | 77.4 |
| Biliary Atresia 14 | Male | 82 | 208 | 308 | 443 | 134.7 | 107.5 |
| Biliary Atresia 15 | Male | 39 | 91 | 136 | 775 | 150.1 | 85.8 |
| Biliary Atresia 16 | Female | 53 | 122 | 266 | 375 | 135.1 | 109.8 |
| Biliary Atresia 17 | Female | 74 | 264 | 363 | 598 | 196.8 | 99.7 |
| Biliary Atresia 18 | Female | 38 | 44 | 82 | 289 | 71.3 | 51.4 |
| Biliary Atresia 19 | Female | 59 | 505 | 529 | 247 | 186.5 | 145 |
| Biliary Atresia 20 | Male | 28 | 136.5 | 257.7 | 104 | 152.5 | 73.94 |
| Biliary Atresia 21 | Male | 75 | 223 | 267 | 251 | 166 | 134.1 |
| Biliary Atresia 22 | Female | 70 | 156 | 235 | 1014 | 159.9 | 105.9 |
| Biliary Atresia 23 | Male | 65 | 67.5 | 304.9 | 229 | 126.4 | 46.08 |
| Biliary Atresia 24 | Female | 58 | 377.4 | 574.8 | 223 | 132.5 | 58.21 |
| Biliary Atresia 25 | Female | 14 | 31.1 | 100.8 | 527 | 307.4 | 80.25 |
| Biliary Atresia 26 | Female | 77 | 315 | 304 | 377 | 121.6 | 95.5 |
| Biliary Atresia 27 | Female | 80 | 177 | 298 | 2327 | 152.7 | 110.7 |
| Biliary Atresia 28 | Male | 81 | 141.1 | 183.1 | 499 | 212.5 | 110.65 |
| Biliary Atresia 29 | Female | 76 | 296 | 496 | 120 | 303.7 | 182.6 |
| Biliary Atresia 30 | Male | 30 | 59.9 | 109.7 | 201 | 219.7 | 60.09 |
| Biliary Atresia 31 | Male | 60 | 77 | 119 | 1877 | 208.7 | 159.7 |
| Biliary Atresia 32 | Female | 60 | 124 | 291 | 707 | 149.2 | 78.5 |
| Biliary Atresia 33 | Male | 60 | 151 | 167 | 203 | 162.7 | 81.2 |
| Biliary Atresia 34 | Female | 60 | 107 | 138 | 177 | 130.8 | 54.7 |
| Biliary Atresia 35 | Female | 60 | 161 | 265 | 261 | 137.5 | 99.5 |
| Biliary Atresia 36 | Male | 60 | 79 | 183 | 223 | 267.3 | 165.4 |
| Biliary Atresia 37 | Female | 60 | 63 | 118 | 321 | 93.9 | 75.2 |
| Biliary Atresia 38 | Male | 60 | 56 | 78 | 101 | 124.5 | 98.2 |
| Biliary Atresia 39 | Male | 60 | 217 | 306 | 1006 | 163.6 | 124 |
| Biliary Atresia 40 | Male | 60 | 12 | 32 | 1225 | 32.1 | 0 |
| Biliary Atresia 41 | Female | 60 | 239 | 569 | 182 | 151.4 | 54.6 |
| Biliary Atresia 42 | Female | 30 | 166 | 155 | 154 | 157.9 | 61.4 |
| Biliary Atresia 43 | Male | 17 | 80 | 133 | 289 | 144.3 | 110.2 |
| Biliary Atresia 44 | Female | 60 | 140 | 286 | 665 | 144.1 | 117.7 |
| Biliary Atresia 45 | Male | 60 | 99 | 158 | 381 | 163.6 | 137.8 |
| Biliary Atresia 46 | Female | 60 | 94 | 141 | 461 | 157.1 | 109.1 |
| Choledochal Cysts 1 | Male | 93 | 12 | 28 | 106 | 191.6 | 0 |
| Choledochal Cysts 2 | Male | 180 | 58 | 61 | 139 | 7.1 | 3 |
| Choledochal Cysts 3 | Male | 60 | 15 | 37 | 52 | 7.3 | 1 |
| Choledochal Cysts 4 | Male | 210 | 215.7 | 131.1 | 948 | 61.9 | 10.18 |
| Choledochal Cysts 5 | Male | 360 | 31.2 | 53 | 29 | 2.2 | 0 |
| Choledochal Cysts 6 | Male | 360 | 28.1 | 47.1 | 59 | 6.6 | 0 |
| Choledochal Cysts 7 | Male | 720 | 13 | 37 | 48 | 3.9 | 1.2 |
| Choledochal Cysts 8 | Male | 120 | 40 | 60 | 57 | 3.5 | 1.9 |
| Choledochal Cysts 9 | Female | 43 | 27.9 | 93.1 | 106 | 229.4 | 0 |
| Choledochal Cysts 10 | Male | 90 | 112 | 111 | 225 | 18.5 | 4.6 |
| Choledochal Cysts 11 | Female | 60 | 176 | 160 | 352 | 10.5 | 4.7 |
| Choledochal Cysts 12 | Female | 90 | 126 | 121 | 21 | 9.2 | 11.5 |
| Choledochal Cysts 13 | Female | 150 | 85 | 29 | 121 | 3 | 12.4 |
| Choledochal Cysts 14 | Male | 90 | 58 | 62 | 64 | 3.1 | 9.3 |
| Atrial Septal Defect 1 | Female | 60 | 85 | 29 | 26 | 6.2 | 5.4 |
| Atrial Septal Defect 2 | Male | 180 | 81 | 34 | 66 | 2.4 | 5 |
| Atrial Septal Defect 3 | Male | 90 | 34 | 38 | 20 | 1.5 | 18 |
| Diaphragmatic Hernia 1 | Male | 300 | 32 | 37 | 11 | 1.5 | ＜1 |
| Diaphragmatic Hernia 2 | Female | 12 | 59.5 | 55.6 | 14 | 7.3 | 0 |
| Hepatoblastoma 1 | Male | 60 | 75 | 89 | 103 | 8.9 | 6.2 |
| Hepatoblastoma 2 | Male | 60 | 88 | 121 | 63 | 3.7 | 2.3 |
| Hepatoblastoma 3 | Female | 90 | 53 | 64 | 64 | 4.6 | 0 |

**Table S2.** **Reagents and Source**

| **Reagent** | **Catalog#** | | **Source** |
| --- | --- | --- | --- |
| HBSS (Ca2+/Mg2+) | G4204-500mL | Servicebio | |
| HBSS (without Ca2+/Mg2+) | G4203-500mL | Servicebio | |
| CollagenaseⅡ | G5029 | Servicebio | |
| Collagenase Ⅳ | G5027 | Servicebio | |
| DNase I | G3342 | Servicebio | |
| CD326 (EpCAM) MicroBeads | 130-105-958 | Miltenyi Biotec | |
| LS column | 130-042-401 | Miltenyi Biotec | |
| Matrigel | 356237 | Corning Biocoat | |
| Advanced DMEM/F12 | G4612-500mL | Servicebio | |
| Penicillin/Streptomycin | 15140-122 | Life Technologies | |
| HEPES | 15630-080 | Life Technologies | |
| B27 | 17504-044 | Gibco | |
| N2 | 17502-048 | Gibco | |
| EGF | PMG8041 | Invitrogen | |
| FGF10 | 100-26-25UG | Pepro tech | |
| L-WRN cells | CRL-3276 | ATCC | |
| D-2-Hydroxyglutaric acid disodium salt | MB7207 | Meilunbio | |
| RNAprep Pure Micro Kit | DP420 | TIANGEN | |
| Hifair® II 1st Strand cDNA Synthesis SuperMix | 11120ES | Yeasen | |
| Hieff® qPCR SYBR Green Master Mix | 11201ES | Yeasen | |
| D-2-Hydroxyglutarate Assay Kit | ab211070, | Abcam | |
| Epigenase 5mC Hydroxylase TET Activity/Inhibition Assay Kit | P-3087 | EpiGentek | |
| Nuclear and Cytoplasmic Protein Extraction Kit | P0027 | Beyotime | |
| Enhanced BCA Protein Assay Kit | P0009 | Beyotime | |
| XF96 cell culture microplates | 101104-004 | Seahorse Biosciences | |
| XF assay medium | 102365-100 | Seahorse Biosciences | |
| XF DMEM medium | 103575-100 | Seahorse Biosciences | |
| Seahorse XF Glycolysis Stress Test Kit | 103020-100 | Seahorse Biosciences | |
| Seahorse XF Cell Mito Stress Test Kit | 103015-100 | Seahorse Biosciences | |

**Table S3. Antibody information**

| Antibody | Source | Catalog# | Application/dilution |
| --- | --- | --- | --- |
| CK19 | Servicebio | GB11197 | IHC /WB (1:500) |
| HNF4α | abcam | ab41898 | IHC/IF (1:200) |
| mTOR | Servicebio | GB111839 | IHC (1:200) |
| Annexin A4 | Servicebio | GB111147 | IF (1:500) |
| CCND1 | Servicebio | GB111372 | IHC(1:1000) |
| ATPB | PROTEINTECH | 17247-1-AP | WB (1:1000) |
| TET1 | Bioss | bs-8523R | IHC (1:200) |
| P-AKT | Cell Signaling Technology | 2965P | WB (1:1000) |
| AKT | Cell Signaling Technology | 4691P | WB (1:1000) |
| 4E-BP1 | Cell Signaling Technology | 9452P | WB (1:1000) |
| Phospho-4E-BP1 (Ser65) | Cell Signaling Technology | 9451P | WB (1:1000) |
| p70(S6K) | PROTEINTECH | 14485-1-AP | WB (1:1000) |
| Phospho-p70 S6K (Thr389) | PROTEINTECH | 28735-1-AP | WB (1:1000) |
| GAPDH | Affinity | AF7021 | WB (1:1500) |
| AMPK | Servicebio | GB112669 | WB (1:1000) |
| Phospho-AMPK(T172) | Bioss | 4002R | WB (1:1000) |
| Phospho-mTOR （S2448） | Cell Signaling Technology | 2976P | WB (1:1000) |
| mTOR | Cell Signaling Technology | 2983P | WB (1:1000) |
| D2HGDH | Proteintech | 66364-1-Ig | WB (1:1000) |
| β-actin | Servicebio | GB11001 | WB (1:1500) |

**Table S4 The primer sequences for qRT-PCR**

| **The sequences of mouse primers** | | | | |
| --- | --- | --- | --- | --- |
| **Genes** |  | **Sequence (5’-3’)** | **Length (bp)** | **Gene ID** |
| Epcam | Forward | AACACAAGACGACGTGGACA | 113 | NM_008532.2 |
|  | Reverse | GCTCTCCGTTCACTCTCAGG |  |  |
| Tacstd2 | Forward | TCACCAAACGGAGGAAGTCG | 108 | NM_020047.3 |
|  | Reverse | GAAGTCGGGGCATCCTACAG |  |  |
| Tjp1 | Forward | TTGTGATACAATACTGTGCCCT | 76 | XM_036152893.1 |
|  | Reverse | GAAATCGTGCTGATGTGCCA |  |  |
| Opa1 | Forward | AGGCCCTTCTCTTGTTAGGT | 99 | NM_001199177.1 |
|  | Reverse | CTTTGTCTGACACCTTCCTGT |  |  |
| Cftr | Forward | GTACGACTCCCTTCAGGCAC | 89 | NM_021050.2 |
|  | Reverse | GGCCCTGGAAGAACCTCATC |  |  |
| Capn1 | Forward | GGGGCTACCGTTTGTCTAGC | 95 | NM_001110504.1 |
|  | Reverse | CTCTGTCATCCTCTGGTGGC |  |  |
| Slc25a3 | Forward | AAGGTATTCACCCCAGGAAAAA | 83 | NM_133668.4 |
|  | Reverse | GGAGTTTCTTAAGTGAGGGGACA |  |  |
| Ucp2 | Forward | GGAAAATCGAGGGGATCGGG | 119 | NM_011671.5 |
|  | Reverse | GGAGTTCTGGAGGCTGCTTT |  |  |
| Ppargc1a | Forward | GCATGAGTGTGTGCTGTGTG | 87 | NM_008904.2 |
|  | Reverse | ACATGTCCCAAGCCATCCAG |  |  |
| Ppara | Forward | TGACGTTTGTGGCTGGTCAA | 113 | NM_001113418.1 |
|  | Reverse | CAGATGGGGCTCTCTGTGTC |  |  |
| Ddit3 | Forward | TATCTTGAGCCTAACACGTCG | 99 | NM_001290183.1 |
|  | Reverse | CCAGGTTCTCTCTCCTCAGGT |  |  |
| Map1lc3b | Forward | GGGACCCTAACCCCATAGGA | 111 | NM_001364358.1 |
|  | Reverse | TCTCCCCCTTGTATCGCTCT |  |  |
| Casp8 | Forward | AAGCAGGAAGTGTGAGAGGC | 106 | NM_001080126.1 |
|  | Reverse | GATCCTCAGGAGGCACCTTG |  |  |
| Krt19 | Forward | AAAACACTGAACCCTGATTCTTG | 94 | NM_001313963.1 |
|  | Reverse | TCTGAAGTCATCTGCAGCCA |  |  |
| Atp5h | Forward | GGGGGTCGGTGAAGTATCC | 87 | NM_027862.1 |
|  | Reverse | GGGGCATGACCTCCACAAAA |  |  |
| Pparg | Forward | CGGGCTGAGAAGTCACGTT | 129 | NM_001127330.3 |
|  | Reverse | TGTGTCAACCATGGTAATTTCAGT |  |  |
| Hnf4a | Forward | CCCTTGGTCATGGTCAGTGT | 71 | NM_001312906.1 |
|  | Reverse | GACCCTGTGAGGGCATAAGG |  |  |
| Gapdh | Forward | CCCTTAAGAGGGATGCTGCC | 124 | NM_001289726.1 |
|  | Reverse | TACGGCCAAATCCGTTCACA |  |  |
| **The sequences of human primers** | | | | |
| D2HGDH | Forward | GGACGTGATCGTGGGCTGTAG | 93 | NM_001287249.2 |
|  | Reverse | AGGTTCCTCTCGTGGCAGTG |  |  |
| HNF4A | Forward | GTGGAGAGTTCTTACGGTGTCA | 108 | XM_047440135.1 |
|  | Reverse | GGGCAGAGGAAGAAAAGCCT |  |  |
| OPA1 | Forward | CTGTGGCCTGGATAGCAGAA | 84 | NM_001354663.2 |
|  | Reverse | AGACTGGCAGACCTCACTCT |  |  |
| CAPN1 | Forward | CTGGATGACCAGATCCAGGC | 118 | NM_001198868.2 |
|  | Reverse | CGCTGATCTCCATGTCCTCC |  |  |
| GAPDH | Forward | GAAAGCCTGCCGGTGACTAA | 150 | NM_001256799.3 |
|  | Reverse | GCCCAATACGACCAAATCAGAGA |  |  |

**Supplementary Figures**

**Figure S1. Hepatic HIF1α increased in BA patients.** (A) Representative immunohistochemistry (IHC) images of HIF1α in livers of BA patients (n = 6) and controls (n = 6). (B) The quantification of panel A. Statistical significance: *****p* < 0.0001.

**
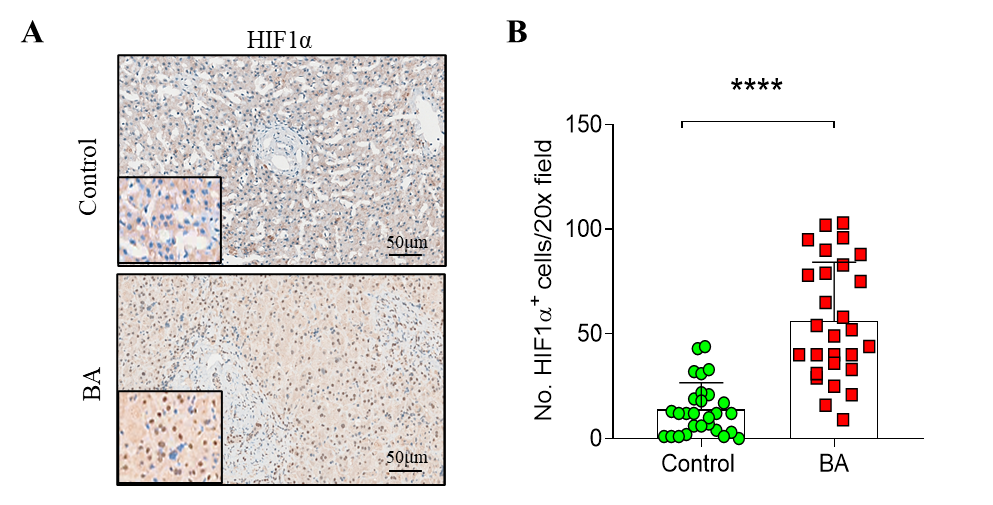
**

**Figure S2. Impaired mTOR signaling in livers of BA patients.** (A) Representative immunohistochemistry (IHC) images of CK19, mTOR and CCND1 in livers of BA patients (n = 6) and controls (n = 6). (B) The quantification of panel A. Statistical significance: **p* < 0.05, ****p* < 0.001.

**
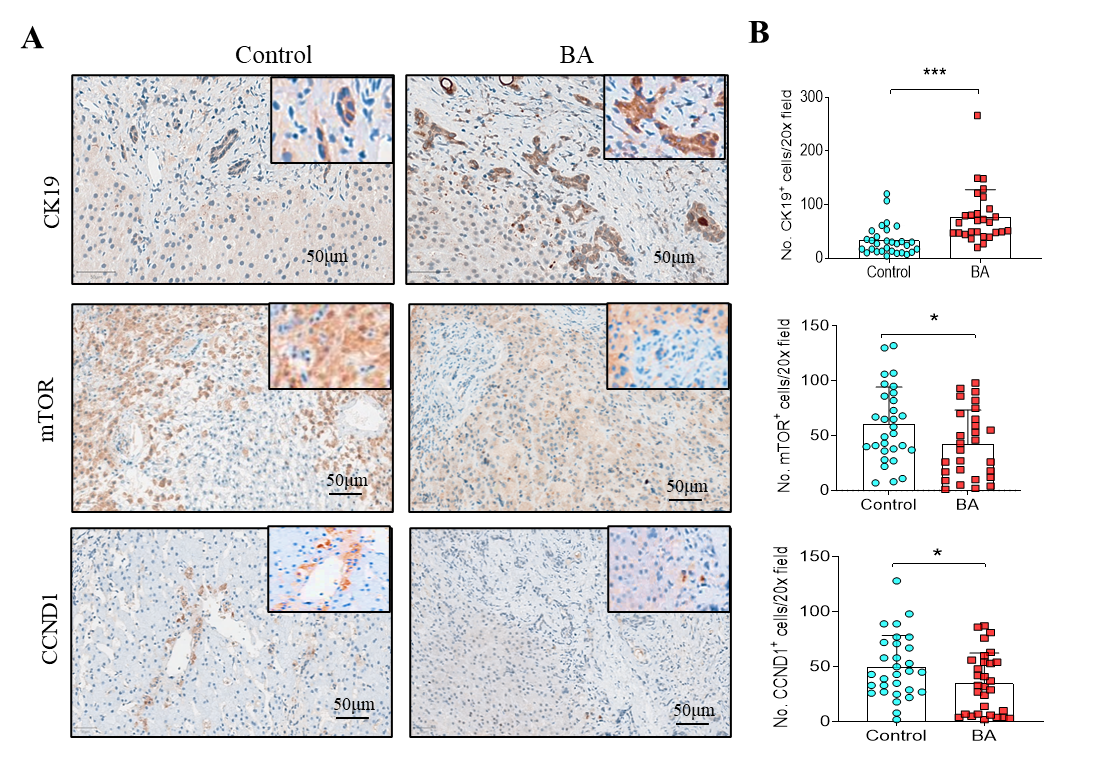
**

**Original and uncropped western-blot bands in this study**

**
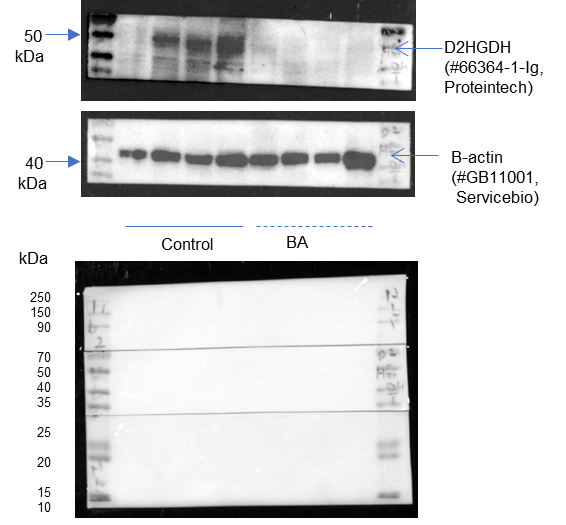
**

**
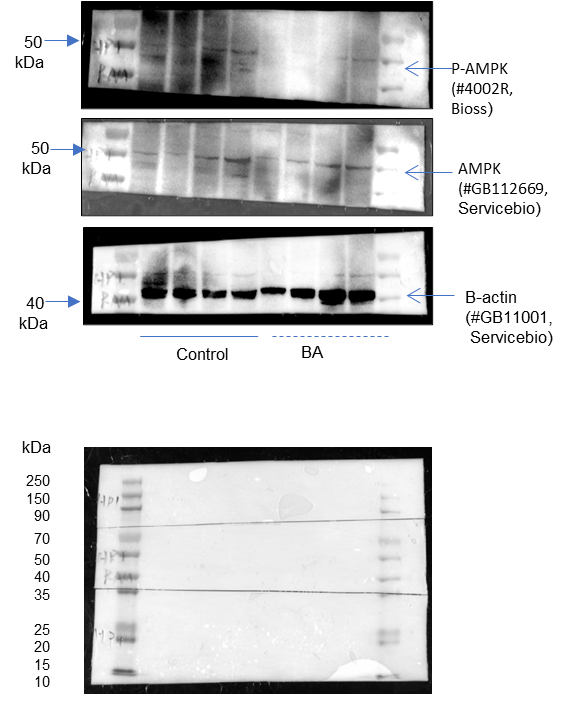
**

**
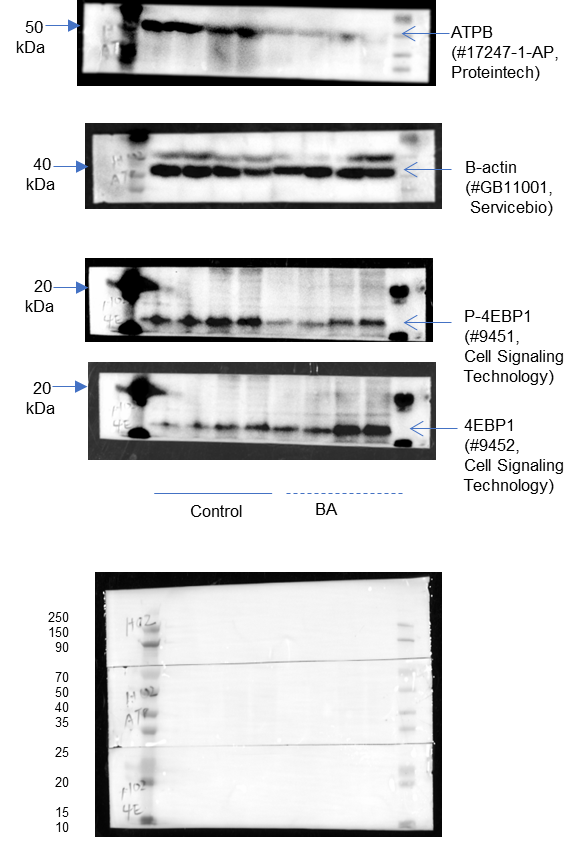
**

**
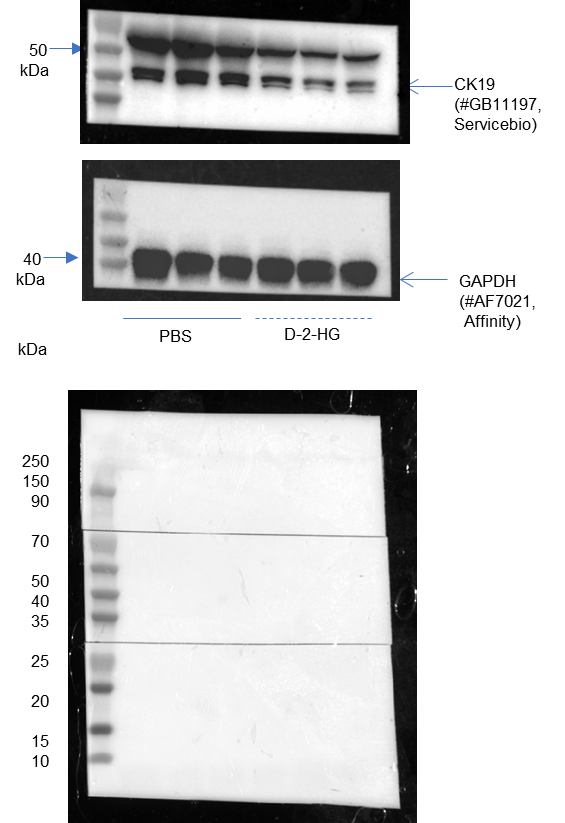
**

**
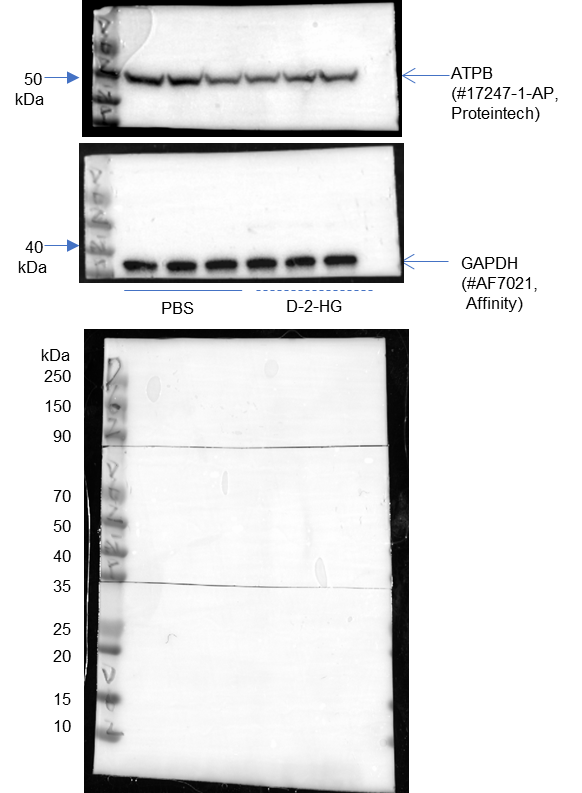
**

**
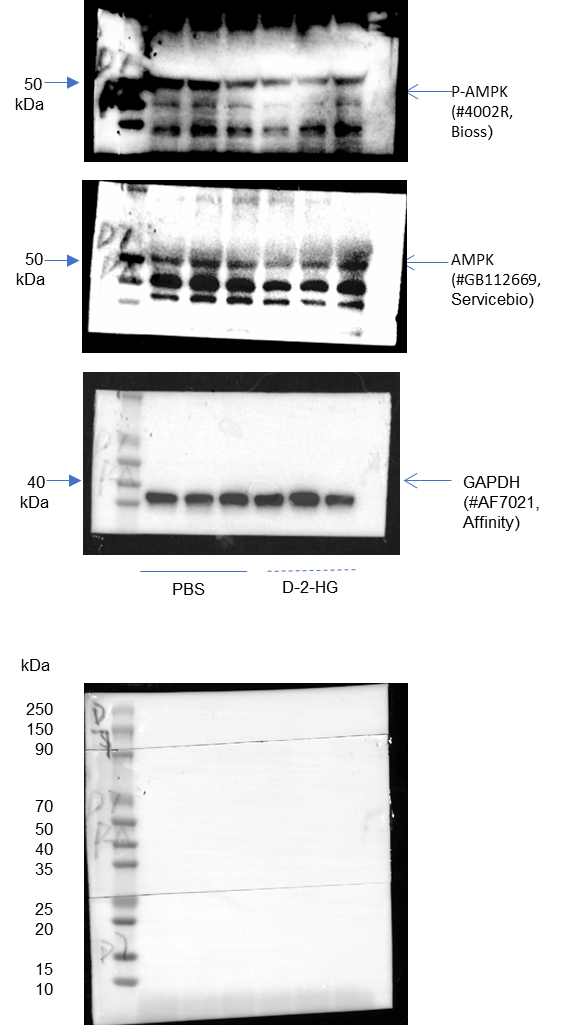
**

**
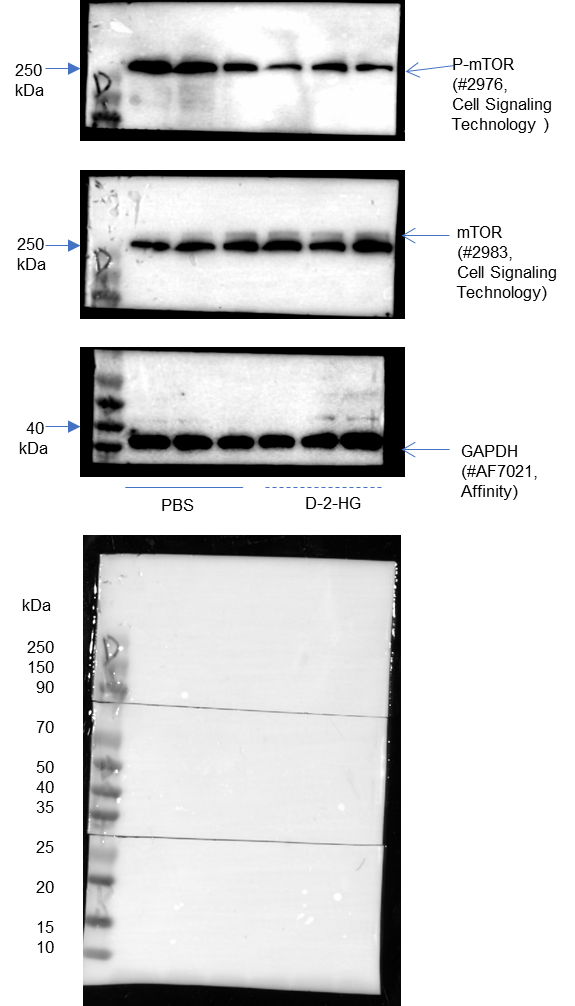
**

**
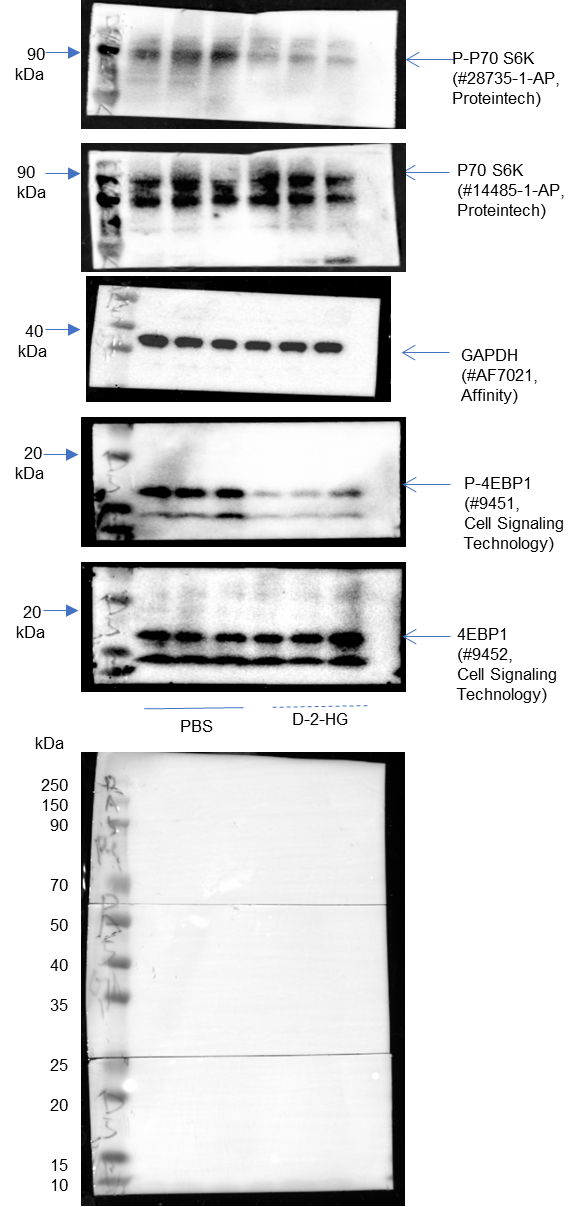

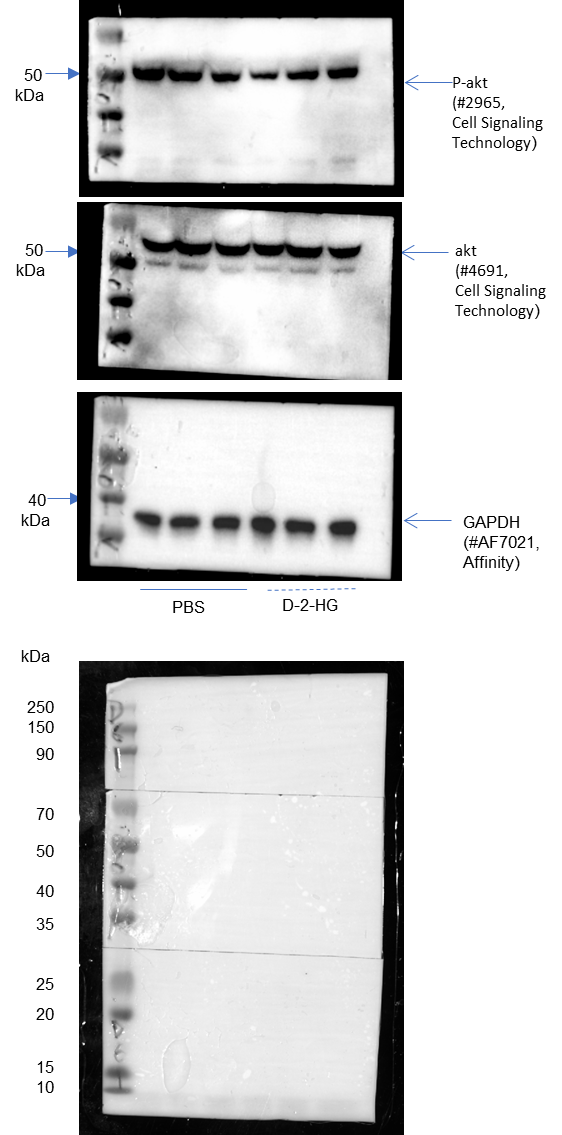
**
